# Measurement of Reduced Gingival Melanosis after Smoking Cessation: A Novel Analysis of Gingival Pigmentation Using Clinical Oral Photographs

**DOI:** 10.3390/ijerph13060598

**Published:** 2016-06-16

**Authors:** Tomotaka Kato, Hiroya Takiuchi, Seiichi Sugiyama, Michiko Makino, Satoshi Noguchi, Tomoko Katayama-Ono, Takashi Hanioka, Toru Naito

**Affiliations:** 1 Section of Geriatric Dentistry, Department of General Dentistry, Fukuoka Dental College, Fukuoka 814-0193, Japan; takiuchi@college.fdcnet.ac.jp (H.T.); makino@college.fdcnet.ac.jp (M.M.); snoguchi@college.fdcnet.ac.jp (S.N.); 2 Sugiyama Dental Clinic, Yachiyo 276-0027, Japan; sdcss@pastel.ocn.ne.jp; 3 Oro-facial Plastic Medical Center, Department of Oral & Maxillofacial Surgery, Fukuoka Dental College, Fukuoka 814-0193, Japan; katat@college.fdcnet.ac.jp; 4 Section of Oral Public Health, Department of Preventive and Public Health Dentistry, Fukuoka Dental College, Fukuoka 814-0193, Japan; haniokat@college.fdcnet.ac.jp

**Keywords:** gingival melanosis, smoking, clinical oral photographs

## Abstract

Background: Due to moisture and the anatomical complexity of the oral mucosa, it is difficult to measure the extent of gingival melanosis in an optical manner. Therefore, we developed a new quantitative method using clinical oral photographs and compared the extent of gingival melanosis before and after smoking cessation. Methods: A new analysis method, which we named the gingival melanosis record (GMR), is a quantitative analysis method using clinical oral photographs. We obtained 659 clinical photographs from 263 patients from 16 general dental offices in Japan. Standardized measuring sites were automatically spotted on the screen, and the presence of gingival melanosis was determined at the measuring sites. We assessed the validity of the GMR with the previously reported Hedin’s classification using Spearman’s rank correlation and intraclass correlation coefficients. Results: The GMR showed a significant association with Hedin’s classification (*p* < 0.01, correlation coefficient = 0.94). The GMR also showed excellent reproducibility of the substantial repeated agreement intraclass correlation coefficients (ICC) (1,1) and ICC (2,1), *p* > 0.61). The longitudinal loss of gingival melanosis was confirmed by a change in the GMR among patients who successfully achieved smoking cessation for a mean of 4.5 years. Conclusion: The GMR is an effective method to assess gingival melanosis. The loss of gingival melanosis after smoking cessation can be objectively confirmed with the use of the GMR.

## 1. Introduction

It is well known that cigarette smoking is a major health problem [1]. Smoking is an apparent risk for periodontal disease [2] and oral cancer [3,4]. Oral healthcare professionals, who have relatively long teachable moments with patients, should work toward developing smoking cessation programs in their own clinics.

In 1977, Hedin *et al.* first reported that smokers showed more pigmented gingiva than non-smokers [5]. They referred to this gingival pigmentation as “smoker’s melanosis”. There is an ethnic difference for gingival melanosis, with a prevalence of 100% in the Black population [6], 40% in the Asian population [7], and 5%–10% in the Caucasian population [8,9]. The pigmentation of the human gingival tissue is derived from melanin granules, which are synthesized in melanosomes of melanocytes [10,11]. The nicotine present in tobacco activates melanocytes to promote melanin secretion [12,13]. Thus, the melanin pigmentation in gingival tissues has a strong correlation with smoking habits [5,14].

A previous study also suggested that the severity of gingival pigmentation decreased after smoking cessation and was related to the number of years after smoking cessation [9,12]. These findings indicate that there is a bi-directional association between tobacco smoking and melanin pigmentation in the gingiva and the possibility of recovery of normal gingival pigmentation by smoking cessation.

Monitoring the gingival color may provide an indicator of the biological reaction to smoking, and may thus be a tool for instruction and supporting smoking cessation. We previously reported the precise measurement of gingival pigmentation with a non-contact type of dental spectrophotometer, and demonstrated the relationship between smoking status and gingival color [15]. However, this procedure requires special equipment. Therefore, we developed a new quantitative method using clinical oral photographs and compared the extent of gingival melanosis before and after smoking cessation with this technique. The purpose of this study was to validate the new quantitative method and to clarify the relationship between smoking cessation and the reduction in gingival melanosis.

## 2. Materials and Methods

### 2.1. Patients and Ethical Considerations

Longitudinal clinical oral photographs and treatment records were obtained with patient consent from 16 general dental offices in Japan. Patients who visited these dental offices for regular check-ups and had series of oral photographs were enrolled in this study. Two hundred and eighty-three patients with 900 oral photographs were enrolled in this study; all patients provided their informed consent. To maintain anonymity, patients were given unique ID numbers and the personal information was kept at the clinic. This study protocol was approved by the Ethics Committee for Clinical Research at Fukuoka Dental College (permission number 194).

### 2.2. Measurement of Gingival Pigmentation

**Figure 1:**
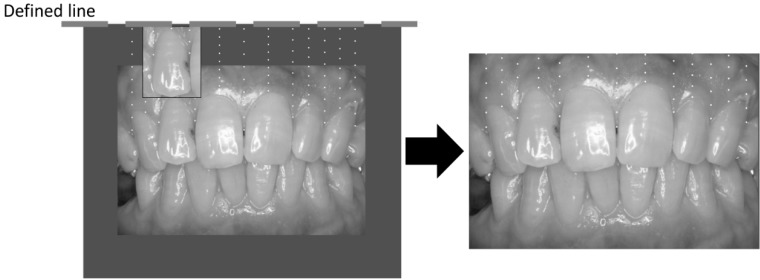
Measurement of gingival pigmentation (gingival melanosis record).

Gingival pigmentation was measured using frontal oral photographs taken by a non-contact-type dental camera. We employed commonly used digital cameras and standardized photograph taking techniques. All the dentists and dental hygienists were took standardized oral photo taking class. Oral photographs were reviewed with a liquid crystal display (Multi Sync LCD 2490WUXi2, NEC Corp., Tokyo, Japan), and the target site was plotted on the screen. The target site was plotted in the following manner (Figure 1): 

(1) A baseline was drawn horizontally on the maxillary gingiva at the crown length of the maxillary right lateral incisor. 

(2) Between the right and left maxillary canines, a vertical line was drawn from the baseline to the cervical line of each tooth. 

(3) Nine points were plotted to separate eight equal parts on the vertical line, and it was adopted that measuring sites were the points in the attached gingiva. To mark the reference mesh on the attached gingiva, we employed Adobe Illustrator. Horizontal lines were placed using the length of the lateral incisor, and divided into nine points, and the vertical lines were placed at mesial and central and distal points of the frontal teeth. These mesh-generating procedures were done with a batch system to avoid the technical assistant's bias and obtain measurement reliability.

(4) The presence of pigmentation at the measuring sites was evaluated and the percentile with pigmentation was calculated for the gingival melanosis record (GMR).

To quantitatively investigate the effect of smoking cessation on gingival melanosis, the patients were divided into the current smoker group and the smoking cessation group. The GMR was used to evaluate the presence or absence of gingival pigment at the assessment sites, and it was represented as a percentage by dividing the pigmented assessment sites by all assessment sites.

### 2.3. Validation of GMR

**Figure 2:**
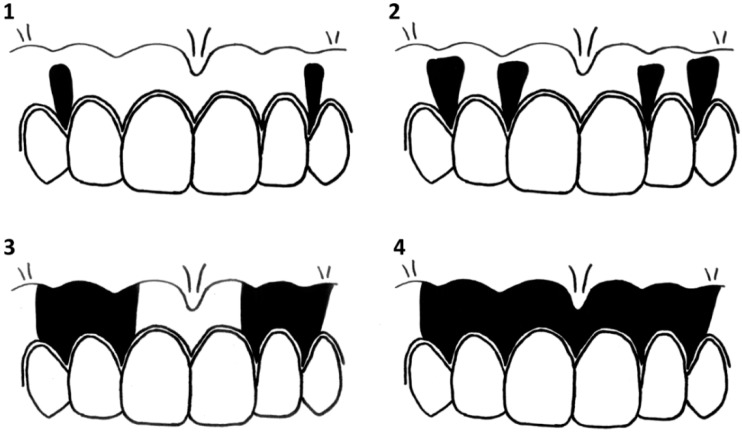
Hedin’s classification (degrees 1–4).

To assess the validity of the GMR, we compared the GMR with Hedin’s classification (Figure 2), which is the gold standard of gingival melanosis classification. In addition, intraclass correlation coefficients (ICC) were calculated to assess the GMR reproducibility. ICC (1,1) was used to evaluate repeatability by the same examiner, and ICC (2,1) was used to evaluate stability between examiners. ICC (1,1) was calculated by the results of one evaluator who performed two analyses with the same 20 oral photographs at a one-week interval. ICC (2,1) was calculated by the results of 20 randomly selected oral photographs using GMR and Hedin’s classification analyzed by three dentists.

### 2.4. Statistical Analysis

Statistical analyses were performed using the IBM SPSS Statistics ver. 22.0 software package (International Business Machines Corp., Armonk, NY, USA). The relationship between the GMR and Hedin’s classification was analyzed using Spearman’s rank correlation coefficient. Differences in the change in the GMR between the current smoker group and the smoking cessation group were analyzed by the Mann-Whitney U test. In addition, the relationship between duration of smoking cessation and the GMR was analyzed with Spearman’s rank correlation coefficient. The significance level was set at 5%.

## 3. Results

### 3.1. Subjects

The characteristics of the study subjects are shown in Table 1. Two hundred and sixty-three patients (mean age 45.9 years; range 19–75 years, 134 current smokers and 129 former smokers) who attended the baseline examination completed this study. Due to unreadable photographs or the absence of routine checkups, 20 patients were excluded in this study. No statistically significant differences between the current smokers and former smokers were observed regarding the baseline characteristics or GMR.

### 3.2. Validation of GMR

**Figure 3:**
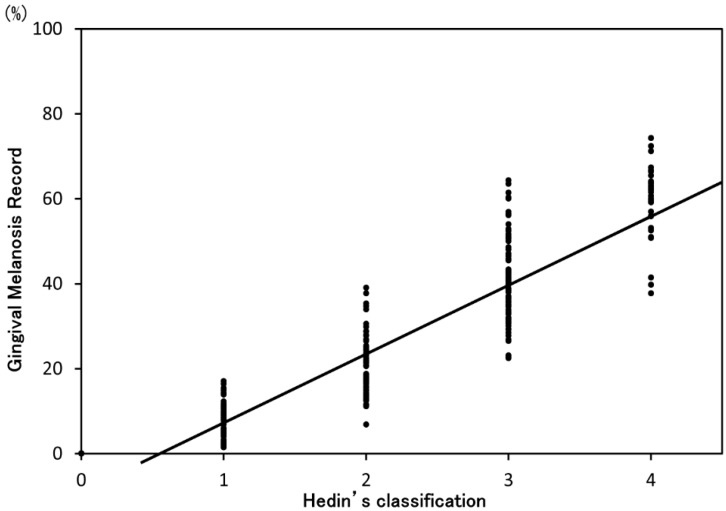
Scatter diagram of the GMR and Hedin’s classification.

Nine hundred photographs were analyzed to evaluate gingival melanosis with the GMR and Hedin’s classification at baseline. Figure 3 shows a scatter diagram of the GMR and Hedin’s classification. A significant correlation was found between the GMR and Hedin’s classification (*p* < 0.01, correlation coefficient = 0.94). For reproducibility, the ICC (1,1) of GMR was 0.781. The ICC (2,1) of GMR (0.783) was higher than that for Hedin’s classification (0.720) (Table 2). Both ICCs for the GMR indicated a “substantial agreement” according to the reference of Landis and Koch [16].

### 3.3. Smoking Cessation and Gingival Melanosis

**Figure 4:**
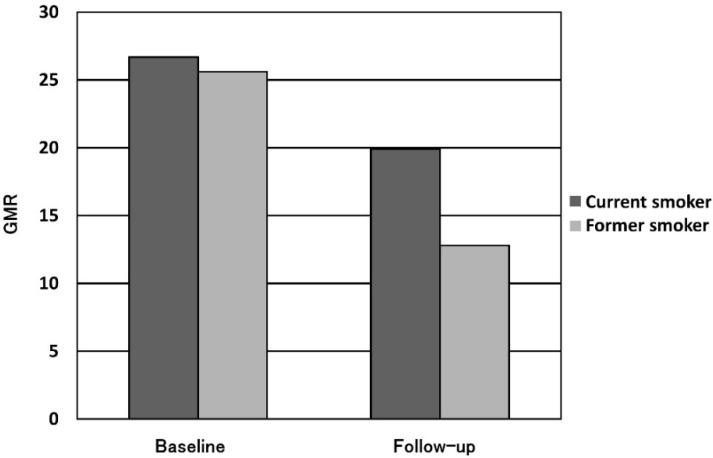
Amount of GMR change (mean GMR for current smoker *vs.* former smoker).

**Figure 5:**
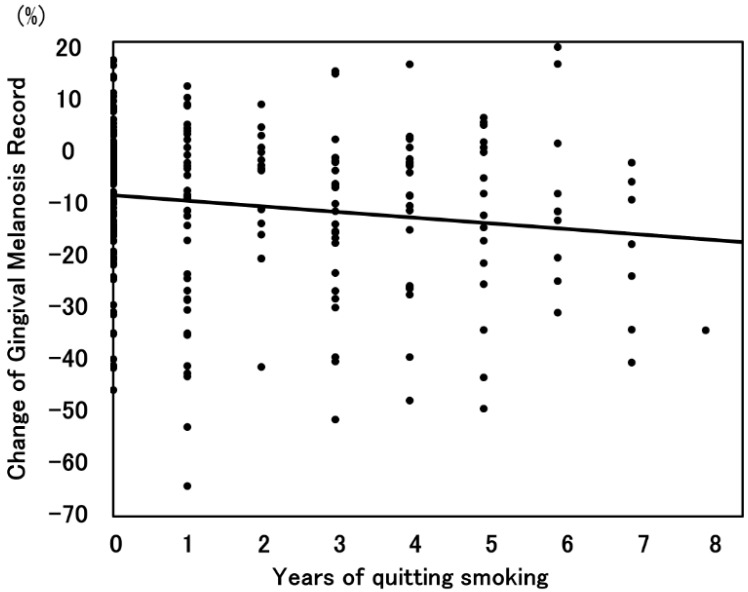
Scatter diagram of the GMR and years of smoking cessation.

The mean follow-up period was 4.5 years for the smoking cessation group and 3.8 years for the current smoking group. The progression of the GMR in each group is shown in Figure 4. The GMR in the current smoking group decreased from 26.7% to 19.9%, and that in the smoking cessation group decreased from 25.6% to 12.8%. A significant difference was observed between the current smoking and smoking cessation groups (*p* < 0.01). There was a significant negative correlation between the reduction in the GMR and the duration of smoking cessation (Figure 5). Additionally, the reduction in the GMR was significantly correlated with the duration of smoking cessation (*p* = 0.01).

## 4. Discussion

In this study, we performed a new quantitative analysis of gingival melanosis using oral photographs. Our quantitative analysis, the GMR, was significantly associated with Hedin’s classification, which is the gold standard for analyzing gingival melanosis. The GMR showed a substantial agreement by ICC (1,1) and ICC (2,1). Additionally, significant differences in the GMR were observed between current smokers and former smokers. Moreover, there was a positive correlation between reductions in the GMR and the duration of smoking cessation. 

Gingival melanosis and its classification were first reported by Hedin *et al.* in 1977 [5]. This classification evaluates gingival melanosis in a subjective manner [17], and other subjective evaluation methods were reported by these authors [9,18,19,20]. Thereafter, quantitative evaluation methods were attempted using a spectrometer or spectrophotometer [21,22,23,24] Recent research has reported a relationship between smoking and gingival melanosis using a spectrophotometer [15]. However, the repeatability of this method is unclear. Moreover, the method requires a particular photometer to evaluate gingival melanosis. In this study, we used oral photographs to evaluate the GMR. In addition, we demonstrated that the GMR was significantly associated with Hedin’s classification and the GMR had a high repeatability to evaluate gingival melanosis. Therefore, we suggest that the GMR is a versatile, ideal method for objective analysis of gingival melanosis as compared to a subjective analysis method such as Hedin’s classification.

Hedin *et al.* reported the relationship between smoking and gingival melanosis and demonstrated that smoking cessation resulted in a reduction in gingival melanosis [12]. Specifically, smoking cessation resulted in a reduction within a few months and gingival melanosis disappeared within six months. However, their studies used subjective methods to evaluate gingival melanosis. Additionally, the amount of melanosis reduction was unclear, and the effect of smoking cessation in smokers could not be evaluated due to the lack of a longitudinal study. In addition, it was a synchronic study in which a quantitative investigation about the relationship between smoking and gingival melanosis was carried out using a spectrophotometer [15]. 

In the present study, we conducted a longitudinal study which compared current smokers to former smokers, and the two groups were similar at baseline. Thus, the effect of smoking cessation was quantitatively evaluated. Few investigations have reported the relationship between the duration of smoking cessation and gingival melanosis in a quantitative manner. Hedin *et al.* reported the relationship between the duration of smoking cessation and oral melanin pigmentation using a subjective analysis [12]. They reported that smoking cessation resulted in a reduction within a few months and gingival melanosis disappeared by six months of smoking cessation. Additionally, they demonstrated a negative correlation between the GMR and the duration of smoking cessation, and considered that 4.5 years of smoking cessation reduced gingival melanosis by half. The study reported by Hedin [12] was a synchronic study, and they measured only the presence or absence of the oral melanosis using a special chart subjectively. In addition, they evaluated the gingiva, buccal mucosa, palate and lip. For these reasons, our study differed from the study of Hedin *et al.*

In the present study, it was interesting that the GMR decreased in the current smoker group and gingival melanosis demonstrated an improvement. Previous studies reported that the quantity of smoking was correlated with gingival melanosis. Araki *et al.* reported that the prevalence of gingival melanosis was significantly higher in smokers who smoked more than 10 cigarettes per day [14]. Additionally, the absence of oral melanosis was recognized in a patient who initially smoked four cheroots per day but reduced the amount to one cheroot per day for nine years [12]. Taken together, these findings suggest that the quantity of smoking reduction might lead to a reduction in gingival melanosis. For future studies, the effect of smoking amount on gingival melanosis should be evaluated.

## 5. Conclusions

The GMR indicated that smoking cessation can reduce gingival melanosis and may be an ideal quantitative analysis for gingival melanosis.

## Figures and Tables

**Table 1:** Patient demographics at baseline.

	Former Smoker	Current Smoker	Total	*p*-Value
Number of subjects	134	129	263	
Male/Female	89/45	81/47	170/92	0.30
Age (years)	46.8 ± 13.4 *	45.0 ± 12.9 *	45.9 ± 13.2 *	0.60
GMR (%)	25.6 ± 18.6 *	26.7 ± 21.0 *	26.2 ± 19.8 *	0.89

* Mean ± SD.

**Table 2:** Validation of the GMR and Hedin’s classification (ICC (1,1) and ICC (2,1)).

	ICC (1,1) (95% CI *)	ICC (2,1) (95% CI *)
GMR	0.78 (0.53–0.91)	0.78 (0.56–0.90)
Hedin’s classification	0.87 (0.703–0.945)	0.72 (0.37–0.89)

* 95% CI: 95% confidence interval.

## Abbreviations

The following abbreviations are used in this manuscript:

GMR: Gingival melanosis record
ICC: Intraclass correlation coefficients
